# PlantPepDB: A manually curated plant peptide database

**DOI:** 10.1038/s41598-020-59165-2

**Published:** 2020-02-10

**Authors:** Durdam Das, Mohini Jaiswal, Fatima Nazish Khan, Shahzaib Ahamad, Shailesh Kumar

**Affiliations:** Data Science Laboratory, National Institute of Plant Genome Research (NIPGR), Aruna Asaf Ali Marg, New Delhi, 110067 India

**Keywords:** Data mining, Databases

## Abstract

Plants produce an array of peptides as part of their innate defense mechanism against pathogens. The potential use of these peptides for various therapeutic purposes is increasing per diem. In order to excel in this research, the community requires web repositories that provide reliable and accurate information about these phyto-peptides. This work is an attempt to bridge the gaps in plant-based peptide research. PlantPepDB is a manually curated database that consists of 3848 plant-derived peptides among which 2821 are experimentally validated at the protein level, 458 have experimental evidence at the transcript level, 530 are predicted and only 39 peptides are inferred from homology. Incorporation of physicochemical properties and tertiary structure into PlantPepDB will help the users to study the therapeutic potential of a peptide, thus, debuts as a powerful resource for therapeutic research. Different options like Simple, Advanced, PhysicoChem and AA composition search along with browsing utilities are provided in the database for the users to execute dynamic search and retrieve the desired data. Interestingly, many peptides that were considered to possess only a single property were found to exhibit multiple properties after careful curation and merging the duplicate data that was collected from published literature and already available databases. Overall, PlantPepDB is the first database comprising detailed analysis and comprehensive information of phyto-peptides from a broad functional range which will be useful for peptide-based applied research. PlantPepDB is freely available at http://www.nipgr.ac.in/PlantPepDB/.

## Introduction

The past decade has seen exceptional growth in peptide-based therapeutic research. Currently, over 60 peptide drugs are approved in the market^1^ and more than 200 peptide drugs are in different clinical trial phases^2^. These numbers clearly denote the applicability of peptide-based therapeutics in the field of drug discovery^3^. Higher as well as the lower group of plants possess a broad range of defense mechanisms to combat chemical, physical and biological stress conditions. Plant-derived peptides are one of their defensive approaches. Bioactive plant peptides are an underexplored domain in the field of proteomics and peptidomics^4^. Plenty of bioactive peptides, including toxins and venoms that act upon intriguing molecular targets, have been identified in all plant taxonomic groups. Plant-derived peptides possess numerous activities like antifungal, antibacterial, antiviral, anticancer, antihypertensive, immune system related, antiparasitic, antifeedant, insecticidal, etc., that can be utilized for many therapeutic and biological applications. Over the last few years, peptides emerged as an alternative for chemical drugs due to the change in drug development and treatment paradigms^1^. Therapeutic peptides are advantageous over proteins or antibodies as they have high target specificity and selectivity as well as easy to synthesize^5^, and are less toxic. Plant peptides may be a starting point for new therapeutic peptides. Apart from therapeutic peptides, there are seven other functional categories of peptides available in PlantPepDB like inhibitory peptides, toxic, plant defense response, microbe killing, etc. which also plays a vital role in several biological phenomena. To achieve more success in plant peptide therapeutics and biological applications, a deeper knowledge of peptide sequence, their complete details are required and a compiled repository of them is more useful. Despite the huge potential of plant peptides, to date, there is no dedicated database available for plant peptides with a variety of therapeutic and bioactive properties. There are various databases available for peptides, such as APD3^6^ (database of antimicrobial, antiparasitic and insecticidal peptides), PhytAMP^7^ (database dedicated to solely antimicrobial plant peptides), Defensins knowledgebase^8^ (devoted to defensin family of antimicrobial peptides), AHTPDB^9^ (database of antihypertensive peptides), BIOPEP^10^ (repository of sensory peptides and amino acids), BaAMPs^11^ (first archive dedicated to biofilm-active antimicrobial peptides), DBAASP^12^ (database of antimicrobial activity and structure of peptides), EROP-Moscow^13^ (oligopeptide sequence database of various activities), LAMP^14^ (database linking antimicrobial, antiparasitic and antitumor peptides), CyBase^15^ (repository of cyclic protein sequences and their structures) and CAMP^16^ (collection of antibacterial, antifungal, antiviral, antiparasitic and certain unclassified peptides). Out of those, only PhytAMP is the plant peptide database, but having only antimicrobial peptides.

**Figure 1:**
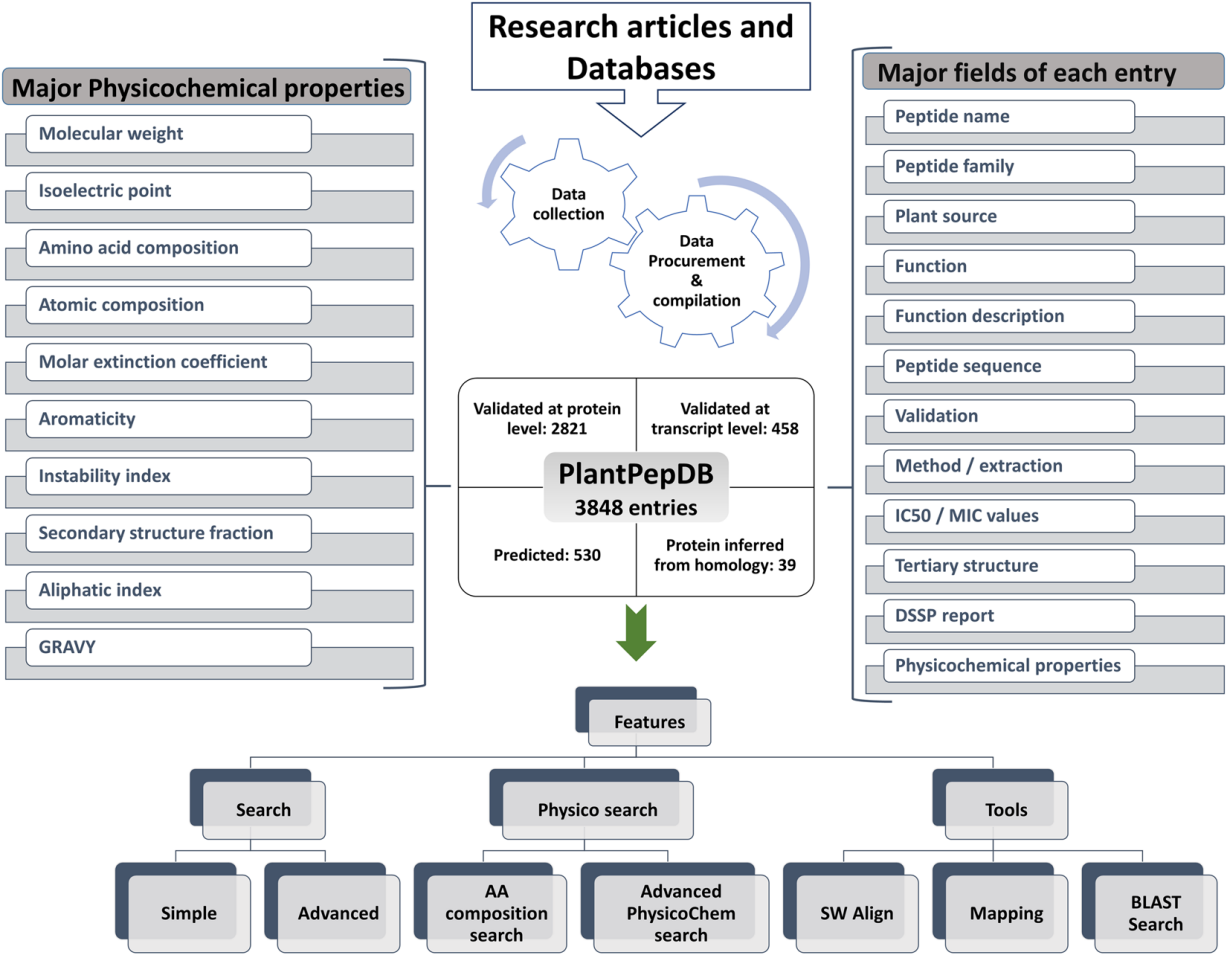
Overall architecture and features of PlantPepDB database.

In this study, we present a comprehensive plant peptide database e.g. PlantPepDB, which provides thorough information about various plant peptides having different functional, physicochemical and structural properties. Overall architecture of PlantPepDB along with its features is displayed in Fig. 1. It consists of 3848 peptide entries collected from 11 different databases and 835 research articles. Peptide sequences from 443 plants have been extracted, out of which only six belong to algae and rest are land plants which include bryophytes, gymnosperms, monocots, and dicots. This database will not only facilitate the plant researchers but will also attract people from a wide range of domain working on peptide-based research.

## Results

### Functional classification of peptides

Based on the curated information of peptide functions, we categorized all the peptide entries into 9 major functional categories. Maximum number of peptides were found in microbe killing functional group with 2356 entries, followed by therapeutic peptides with 1465 entries, toxic peptides (407 entries), inhibitory peptides (280 entries), invertebrate killing (209 entries), immune system related (65 entries), plant defense response (43 entries) and opioid peptides (8 entries). A separate category named as miscellaneous with 231 peptides is also created for those functional categories that do not fit into any other category. The therapeutic functional category consists of 31 diverse classes of peptides, like anticancer, vasorelaxant, anti-hypertensive, diuretic, antithrombotic, anti-inflammatory, antidiabetic, etc. Along with functional and sub-functional classification of peptides, their responses in organisms are also mentioned i.e. whether the peptides will exhibit their response in plants, animals or other organisms. Detailed information about the number of peptides present in each functional and sub-functional category along with their response information is illustrated in Table 1.

**Table 1:** List of functional and sub-functional category of peptides along with their response information incorporated in PlantPepDB.

Functional Category	Number of Peptides	Sub-functional Category	Response in Plants/Animal/Others
Inhibitory in nature	280	Protein translation inhibitor (2), Enzyme inhibitor (256), Protease inhibitor (13), Serine protease inhibitor (1), Tyrosinase and melanin inhibitor (3), Tyrosinase inhibitor (5)	Animal
Toxic	407	Toxin (37), Celiac toxic (201), Cytotoxic (169)	Animal
Immune system related	65	Immunomodulatory (35), Immunoregulator (10), Immunostimulating (1), Immunosuppressive (19)	Animal
Opioid	8	Opioid (6), Opioid agonist (1), Opioid antagonist (1)	Animal
Therapeutic	1465	Antiproliferative (9), Anticancer (156), Vasorelaxant (2), Antihypertensive (500), ACE-inhibitor (427), Hypotensive (5), Pore-forming (1), Antithrombotic (7), Antioxidant (227), Anti-inflammatory (13), Anti-amnestic (4), Anti-analgesic (1), Antinociceptive (3), Anxiolytic (2), Diuretic (2), Uterotonic (1), Anti-HIV (37), HIV-1-reverse-transcriptase inhibition (8), Antihyperglycemic (1), Antidiabetic (1), Hypoglycemic (1), DPP-IV inhibitor (3), Estrogen like activity (18), Phagocytosis stimulatory peptide (2), Bile acid binding inhibitor (1), Protein synthesis inhibitor (2), Cyclooxygenase inhibitor (8), HMG-CoA reductase inhibitor (3), Neurotensin inhibitor (1), Anti-allergen (11), Antimalarial (8)	Animal
Plant defense response	43	Alpha-amylase inhibitor (15), Defensive-proteinase inhibitor (3), Trypsin inhibitor (3), Gene expression activator (5), Gene expression stimulator (1), Antifeedant (7), Defense activator (3), Defense gene activator (6)	Plants
Microbe killing	2356	Antimicrobial (1393), Antiparasitic (15), Antiprotist (4), Antibacterial (323), Antiyeast (4), Antifungal (529), Antibiotic (2), Antiviral (83), Antibiofilm (3)	Others
Invertebrate killing	209	Anthelmintic (35), Anti-barnacle (1), Molluscicidal (6), Nematocide (56), Insecticidal (111)	Animal
Miscellaneous	231	Hemolytic (71), Hypocholesterolemic (2), Hypotriglyceridemic (3), Neuropeptide (92), Allergen (9), Enzymatic degradation (54)	Animal

### Sequence length, plant source and functional relationship of plant peptides in PlantPepDB

Currently, there are 3848 unique peptide entries that have been incorporated in the database from 11 different published databases and 835 published literature articles. The database consists of peptides of varying length range. There are 2862 peptides which are less than or equal to 50 residues in length (i.e. about 75%) and rest 986 peptides are of length above 50 residues (i.e. only about 25%). The database consists of peptides extracted from 443 plants among which the maximum number of peptides were found in Glycine max with 296 peptides followed by Arabidopsis thaliana with 150 peptides and Triticum aestivum with 142 peptides.

It was also seen that many peptides despite been collected from different resources exhibit multiple properties. Out of 3848 peptides, 2673 exhibit single function, however, 556 peptides exhibit two functions, 239 show three functions, 69 peptides show four functions, 30 exhibits five functions, and only 19 peptides exhibit more than five functions. The peptides which are found to display multiple functions may play vital roles in various therapeutic and biological activities. Much detailed information about the peptides which were found to possess more than five functions is shown in Table 2. The information of each of those 19 peptides can be tracked by using the PPepDB ID given in the table. All of these peptides are available in multiple sources but the information was very scattered till we collected all the data, curated it and compiled it and incorporated in PlantPepDB. We have also incorporated information about peptide families, among which cyclotides was the most occurring peptide family with 27.4% of the total peptides, followed by thaumatin peptide family with 26.5%, defensin family with 10.7% and rest all the families had below 10% of peptides like thionin (6.7%), ACE inhibitory peptide (6.5%), Orbitide (4.6%), lipid-transfer (2%), hevein family (1.5%), glycinin (2.2%), snaking (1.1%), cyclic peptides (0.7%) and lectin family (0.6%). There are other peptide families also which comprise a very small number of peptides and hence collectively put into the “other” category (8.1%). A detailed pie chart is available in the statistics page of PlantPepDB.

**Table 2:** List of peptides which are showing more than five activities along with their PPepDB.

PPepDB ID	Data source	Peptide name	Functions	Sequence	Length
PPepDB_1570	CAMP, Cybase, EROP-Moscow	Cter L	Antimicrobial, Insecticidal, Hemolytic, Anthelmintic, Antibacterial, Cytotoxic	HEPCGESCVFIPCITTVVGCSCKNKVCYD	29
PPepDB_1571	CAMP, Cybase, EROP-Moscow	Cter K	Antimicrobial, Insecticidal, Hemolytic, Anthelmintic, Antibacterial, Cytotoxic	HEPCGESCVFIPCITTVVGCSCKNKVCYN	29
PPepDB_1925	Cybase, APD, CAMP, EROP-Moscow, LAMP, PhytAMP	Cyclotoviolacin O15	Nematocide, Hemolytic, Antibacterial, Antifungal, Antiviral, Antiparasitic	GLVPCGETCFTGKCYTPGCSCSYPICKKN	29
PPepDB_1926	Cybase, APD, CAMP, EROP-Moscow, LAMP, PhytAMP	Cycloviolacin O14	Nematocide, Anti-HIV, Hemolytic, Enzymatic-degradation, Antibacterial, Antifungal, Antiviral, Anti-HIV, Antiparasitic	GSIPACGESCFKGKCYTPGCSCSKYPLCAKN	31
PPepDB_2070	DBAASP, APD, Cybase, Literature	Cyclotide Cter M, Cyclotide cliotide T3	Anticancer, Insecticidal, Hemolytic, Anthelmintic, Antibacterial, Cytotoxic	GLPTCGETCTLGTCYVPDCSCSWPICMKN	29
PPepDB_2096	DBAASP, CAMP, APD, Cybase	Cyclotide cter-P, Cyclotide cliotide T4	Anticancer, Insecticidal, Hemolytic, Anthelmintic, Antibacterial, Cytotoxic	GIPCGESCVFIPCITAAIGCSCKSKVCYRN	30
PPepDB_2104	DBAASP, CAMP, Literature	Coccinin	Antiviral, Anticancer, Antifungal, Hemolytic, Antiproliferative, HIV-1-reverse-transcriptase-inhibition	KQTENLADTY	10
PPepDB_2126	DBAASP, Cybase, CAMP, APD, Literature	Cliotide T1	Antibacterial, Anticancer, Cytotoxic, Antimicrobial, Immunomodulatory, Nematocide, Hemolytic	GIPCGESCVFIPCITGAIGCSCKSKVCYRN	30
PPepDB_2160	DBAASP, EROP-Moscow, CAMP, Cybase	Cter G	Antibacterial, Anticancer, Antifungal, Insecticidal, Hemolytic, Anthelmintic, Cytotoxic	GLPCGESCVFIPCITTVVGCSCKNKVCYNN	30
PPepDB_2170	DBAASP, EROP-Moscow, Cybase, CAMP, APD	Tricyclon-A	Antibacterial, Anticancer, Antifungal, Antiviral, Hemolytic, Antimicrobial	GGTIFDCGESCFLGTCYTKGCSCGEWKLCYGTN	33
PPepDB_2207	DBAASP, PhytAMP, EROP-Moscow, Cybase, APD, Literature	Kalata B2	Antibacterial, Anticancer, Antifungal, Nematocide, Molluscicidal, Insecticidal, Hemolytic, Antiviral, Antiparasitic	GLPVCGETCFGGTCNTPGCSCTWPICTRD	29
PPepDB_2211	DBAASP, PhytAMP, EROP-Moscow, Cybase, CAMP, APD	Kalata B7	Antibacterial, Anticancer, Antifungal, Nematocide, Molluscicidal, Antiparasitic, Hemolytic, Antimicrobial	GLPVCGETCTLGTCYTQGCTCSWPICKRN	29
PPepDB_2214	DBAASP, PhytAMP, EROP-Moscow, Cybase, CAMP, APD	Kalata B1	Antibacterial, Antifungal, Antiviral, Anticancer, Hemolytic, Cytotoxic, Nematocide, Molluscicidal, Insecticidal, Enzymatic-degradation, Anti-HIV, Enzyme-inhibitor	GLPVCGETCVGGTCNTPGCTCSWPVCTRN	29
PPepDB_2215	DBAASP, PhytAMP, EROP-Moscow, Cybase, CAMP, APD	Circulin-B, CIRB	Antibacterial, Antifungal, Hemolytic, Cytotoxic, Antiviral, Insecticidal, Anti-HIV	GVIPCGESCVFIPCISTLLGCSCKNKVCYRN	31
PPepDB_2226	DBAASP, PhytAMP, LAMP, EROP-Moscow, Cybase, CAMP, APD, Literature	Cycloviolacin-O2	Antibacterial, Anticancer, Antifungal, Cytotoxic, Nematocide, Hemolytic, Anti-barnacle, Antiparasitic, Antimicrobial	GIPCGESCVWIPCISSAIGCSCKSKVCYRN	30
PPepDB_3844	Literature, EROP-Moscow, BIOPEP	Oryzatensin	Anti-analgesic, Anti-amnestic, Anticancer, Immunomodulatory, Neuropeptide, Opioid-antagonist	GYPMYPLPR	9
PPepDB_3859	Literature, PhytAMP, EROP-Moscow, Cybase, CAMP, APD	Varv-A	Cytotoxic, Nematocide, Hemolytic, Anti-HIV, Anticancer, Antimicrobial	GLPVCGETCVGGTCNTPGCSCSWPVCTRN	29
PPepDB_3860	Literature, PhytAMP, LAMP, EROP-Moscow, Cybase, CAMP, APD	Cyclovialacin O24	Antibacterial, Antifungal, Antiviral, Nematocide, Anti-HIV, Hemolytic, Enzymatic-degradation	GLPTCGETCFGGTCNTPGCTCDPWPVCTHN	30
PPepDB_3992	PhytAMP, LAMP, EROP-Moscow, CAMP, APD, Cybase	Cycloviolacin-O13 (Cyclotide c3)	Nematocide, Anti-HIV, Hemolytic, Enzymatic-degradation, Antibacterial, Antifungal, Antiviral, Antiparasitic	GIPCGESCVWIPCISAAIGCSCKSKVCYRN	30

ID, source of data, peptide name, functions, sequence and sequence length.

### Peptide structural statistics

**Figure 2:**
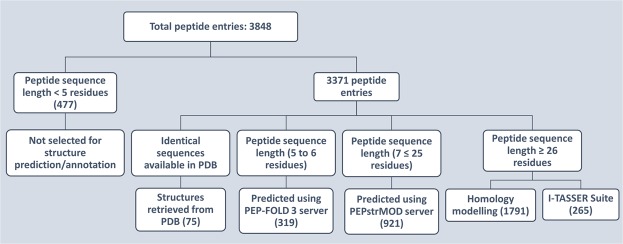
Schematic representation of structural annotation steps used to model the tertiary structure of peptides of PlantPepDB.

The peptides which have a minimum length of 5 residues were modelled using various modelling approaches. The distribution of peptide structures across the modelling tools that were used can be found in Fig. 2. First, there were 477 peptides whose sequence length was below 5 residues, and these were not considered for structural modelling. There were 75 peptides for which the tertiary structures were already available, and were extracted from PDB database. The peptides with length 5 and 6 residues were modelled using PEP-FOLD 3^17^ server which is a server for de novo peptide structure prediction. A total of 319 peptides were modelled using this server which had the sequence length of 5 and 6 residues. A total of 921 peptides with sequence length from 7 to 25 residues were modelled using PEPstrMOD^18^ (in batch mode) webserver, a state-of-art method used to predict the tertiary structure of peptides. Out of 2056 remaining peptides, 1791 peptides were modelled using homology modelling approach since all of them had significant homologs available in the PDB database. For all these 1791 structures, 5 models were built using the best template and out of them, the model with the least DOPE score was selected as the final model. Finally, the remaining 265 peptide sequences that had no significant homolog in PDB were modelled using I-TASSER Suite^19^ which is considered to be the gold standard tool for de novo modelling of protein tertiary structures. DSSP software package was used to assign the secondary structural states of all the peptides available at PlantPepDB. The tertiary structures of all the peptides were given as input to the DSSP except 477 peptide entries whose structure was not modelled due to sequence length below 5 residues. The DSSP output reports of each peptide in PlantPepDB is available in the database along with the tertiary structure.

### Utility of PlantPepDB

PlantPepDB is a powerful web-resource that provides in-depth information about most of the bioactive and therapeutic plant peptides published so far. The physicochemical properties and the structure of peptides play a vital role in the functional aspect. Both of these information have been incorporated into the database. User can exploit the structural information of peptides for further in-silico screening studies, docking, binding pockets, molecular simulations and peptide interaction with receptors. The biggest advantage of a database like PlantPepDB is that users can get all information of a single peptide, in a single entry. For example, plant peptide with id ‘PPepDB_3988’ is ‘Fabatin-1’ which is present in multiple databases like PhytAMP^7^, EROP-Moscow^13^, DEFENSINS-Knowledgebase^8^, CAMP^16^ and APD3^20^ with various information. Now in PlantPepDB, users can find all those scattered information and additional curated information about ‘Fabatin-1’ in a single place which will prove to be extremely useful. Users can even search peptides based on their physicochemical properties using the ‘Advanced PhysicoChem Search’ module of PlantPepDB. This is very useful in case users have pre-handed information about their experiments and they want plant peptides with particular properties.

## Discussion

Bioactive and therapeutic peptides comprise of a wide class of peptides having biological activities. The discovery of insulin therapy in 1920s has led peptide therapeutics to play a vital role in medical practice. Diversification has occurred in peptide drug discovery to integrate a broad array of structures noted from diverse natural sources or via efforts of medicinal chemistry, apart from its conventional focus on endogenous human peptides. Peptides are molecularly poised amid proteins and small molecules, even so therapeutically and biochemically dissimilar from two of them, thus represents an exclusive category of pharmaceutical compounds. By engineering enhanced pharmaceutical properties, by utilizing strategies of novel chemistry for broadening molecular diversity and by developing into new indications and molecular targets, the pace has been maintained by peptide therapeutics with scientific innovation. To facilitate peptide-based research, several peptide databases are available which contain peptides of many functional categories, however, there are no peptide databases dedicated to peptides extracted from plant sources except PhytAMP which only contains 273 peptide entries with limited functional categories. In an attempt to make a comprehensive meta-database, PlantPepDB is developed, embodying a compilation of peptides which are derived from only plant sources with a wide variety of functional categories. PlantPepDB is developed in an attempt to make a comprehensive meta-database comprising a collection of peptides which are derived from only plant sources with a wide variety of functional categories. All the entries present in PlantPepDB are cross-linked with their original sources for easy access to the primary source. It contains peptides from already available databases as well as from several manually curated literature sources. However, collecting from such a huge amount of dataset showed a problem that has been carefully addressed in PlantPepDB which is the number of duplicate entries which are having the same peptide sequence, but different meta-information collected from different sources (both literature and databases). In this database, such peptide entries have been manually curated to merge all the meta-information of a peptide sequence, thus reducing the number of repeated and duplicate peptide entries into a single information enriched entry. Further, the physicochemical properties like molecular mass, isoelectric point, amino acid, and atomic composition, molar extinction coefficient, aromaticity, instability index, Grand Average of hydropathicity and aliphatic index etc. of each and every peptide has been incorporated into the database which will facilitate the user to decide the usability of a peptide in various experimental studies. The tertiary structure of all peptides that have more than 5 residues have been integrated into the database which are vital for screening, docking and simulation studies. Moreover, many peptides which were described in different sources with single functional property were found to possess multiple properties when repeated and duplicate peptide sequences were merged, which make PlantPepDB a very effective web resource of plant peptides.

In a nutshell, users can take advantage of PlantPepDB in the following means: (i) searching for plant peptides from 11 databases and 835 research articles at one go and therefore save time, (ii) with all the additionally curated meta-data that is available in the database, users can find much more comprehensive information than available in the primary sources, (iii) users can search the data by amino acid composition as well as by setting various physicochemical property parameters, (iv) extract structural information for most of the peptides with 5 or more residues, that will facilitate structure to function analysis as well as screening and docking studies. We hope that the development of PlantPepDB will expedite the plant peptide research.

## Methods

### Data collection

The data was collected from an extensive literature search as well as from currently available peptide databases. The majority of the data of PlantPepDB was acquired from 11 databases: AHTPDB^9^, APD3^20^, BIOPEP^10^, PhytAMP^7^, EROP-Moscow^13^, Defensins knowledgebase^8^, BaAMPs^11^, LAMP^14^, Cybase^15^, CAMP^16^, and DBAASP^12^. The entries from databases were first filtered for plant peptides only. Data retrieval was done using export and download options available at the databases. In case, there is no option to download the data, we used our in-house Perl scripts to retrieve the data and, in some cases, used ‘wget’ command. Using ‘wget’ and Perl scripts, we have downloaded the information of peptides in ‘HTML’ format from the databases, which were processed using Linux commands and ‘awk’ scripts to extract desired information. A total of 835 research articles were manually curated for bioactive plant peptides information. Since the literature was very huge to search from, therefore, to narrow down the papers only to search for plant-related peptides, we used the advanced search option of NCBI’s PubMed database. The search for articles was performed by queries using various combinations of keywords (e.g. to search for all the antimicrobial peptides published in the last 5 years of *Arabidopsis thaliana* plant we used keywords like ‘antimicrobial’, ‘peptides’, ‘Arabidopsis thaliana’). Further, research articles and reviews lacking relevant or insufficient information were excluded. Full-text search was performed for all the relevant articles having any plant peptide information and was curated to form a tabular format.

### Curation and compilation of peptides

We curated the functional properties of each peptide from their source database as well as literature. Initially, after collecting and compiling all the data into a tabular format we had 8356 plant peptide entries but after the second level of curation and refinement of the data, we were left with 3848 plant peptide entries. The second level of curation involved regrouping of duplicate and repeated peptide entries and making only one information-rich entry. For e.g.: same peptide information is available in two different databases or articles, but both the sources contain partly different information like one source has information about peptide activity, plant source, activity against two bacteria while the other source also contains the same peptide and most of the reported information is same, but some are different and new information like activity against some fungal infection or shown to possess toxic property. Initially, these two entries were separate but after the second level of manual curation, such entries were merged to form one single data enriched peptide entry. This careful curation will help the researchers to get all the information in a single entry, collected from multiple research articles and databases.

### Structural annotation of peptides

An organized approach was used to implement the structural annotation of all the peptides and this is comprehensively shown in Fig. 2. Initially, all the peptide sequences in the PlantPepDB database were examined for an identical sequence in Protein Data Bank (PDB)^21^. In case, an identical sequence was available in PDB, we retrieved that structure and assigned it to the matching PlantPepDB peptide entry. If the identical sequence was not available in PDB, then we used different pipelines for predicting the structure of peptides depending on the length of peptides. The peptides which were having a sequence length below five were not modelled. The peptides with length five to six residues were modelled using PEP-FOLD3^17^ Server. The peptides with sequence length 7 to 25 were modelled using PEPstrMOD^18^ which is again a peptide structure prediction server. The jobs in PEPstrMOD were submitted for a batch run so that multiple structures can be modelled simultaneously. The peptides with length more than 25 residues having homologous structures in PDB (i.e. sequence identity >40% and sequence query coverage >50%) were predicted using homology modelling. The top templates were used to make the tertiary structure of peptides using MODELLER^22^. Finally, the remaining peptides which did not have any significant homolog in PDB, were modelled via the de-novo approach using I-TASSER Suite^19^ in a parallel manner so that multiple cores can be used to run the modelling jobs quickly. We used DSSP software^23,24^ to assign eight types of secondary structural states (H: alpha helix, G: 3/10 helix, I: pi helix, B: beta-bridge, E: extended strand, S: bend, C: loop and T: turn)^9^ by providing the tertiary structure of peptide in PDB file format as input.

### Physicochemical properties of peptides

All the 3848 peptides of PlantPepDB were analyzed carefully and the physicochemical properties of the peptides were calculated using the peptide sequences. We used biopython module ‘Bio.SeqUtils’^25^ from which we imported the ProtParam utility in which the peptide sequences were provided as inputs. In-house python scripts were used to run 10 types of analysis on peptide sequences, like amino acid count, amino acid percent, isoelectric point, molecular weight, Grand average of hydropathicity (GRAVY) index, aromaticity, instability index, atomic composition, molar extinction coefficient and secondary structure fraction. Some properties like the number of positively and negatively charged residues were calculated by the previously calculated amino acid counts. The aliphatic index of peptides was calculated using the formula given on the Expassy Bioinformatics Resource Portal’s ProtParam^26^ tool documentation i.e. Aliphatic index = X(Ala) + a ∗ X(Val) + b ∗ (X(Ile) + X(Leu)) where X(Ala), X(Val), X(Ile), and X(Leu) are mole percent (100 X mole fraction) of alanine, valine, isoleucine, and leucine. The coefficients a and b are the relative volume of the valine side chain (a = 2.9) and of Leu/Ile side chains (b = 3.9) to the side chain of alanine. The Atomic Composition of peptides which tells us about the carbon, hydrogen, nitrogen, oxygen and sulfur content was calculated using the Proteomics Toolkit (http://db.systemsbiology.net:8080/proteomicsToolkit/IsotopeServlet.html) developed by Institute for Systems Biology. We have used an in-house Perl script to provide automated input to the proteomics toolkit server and extract out the atomic composition information in tabular format.

### PlantPepDB web interface

We have developed PlantPepDB using Apache HTTP server (version 2.4.6) integrated with PHP (version 7.3.3) and MySQL (version 8.0.15) on a server machine with Centos 7 Linux as the operating system. JavaScript (version 1.8.0) and PHP were used to develop the back-end of the database while MySQL (version 8.0.15) was used to process the data at the back-end. CSS and HTML were used to make the template responsive. Perl scripts were also integrated at the back-end of the database for multiple file handling and data manipulation.

### Data retrieval tools

‘Simple Search’ module allows user to search the PlantPepDB database using various keywords from different fields given on the search page. Users can search from one field at a time. To make simple search more flexible, ‘containing’ and ‘exact’ options are also incorporated to search for a wide range of data to a more specific search. In the ‘Advanced search’ module, users can make complex queries and can search from multiple fields using conditional operators for each field. Advanced search allows user to search from 14 different fields that cover all the important information about a peptide entry in PlantPepDB. Users can download the results of the search in both cases.

The previously mentioned search options majorly deal with basic peptide information but the ‘Physico Search’ option allows the user to search peptides based on their physicochemical properties. ‘AA Composition Search’ allows users to search peptides according to their amino acid count as well as amino acid percentage. Users must provide a minimum and maximum range of amino acids to search for peptides. Users can select different amino acids by using the dropdown in the amino acid selection column. The ‘Advanced PhysicoChem Search’ is very similar to ‘advanced search’, however, the only difference is that this search module is completely dedicated to physicochemical properties. There are total of 16 physicochemical properties from which the user can perform the search (e.g. molecular weight, gravy, aliphatic index, instability index, aromaticity, etc.).

### Data browsing in PlantPepDB

The ‘Browse module’ provides 4 major options (e.g. plant classification, peptide family, peptide activity and sequence length range) to browse all the entries of this database. Hence, if a user is interested in extracting all the peptides which belong to the Defensin family, the user have to use ‘Browse Peptide Family → Defensin’ to get the desired results. This module is useful in case a user has very little or no information on what to search on the database. The user can choose from the available options and can obtain all the information on the peptide.

### Tools incorporated in PlantPepDB

We have incorporated a total of three tools at PlantPepDB to facilitate the user to find and match their query peptide sequences with the PlantPepDB sequences. The first tool is ‘SW Align’ i.e. Smith-Waterman Alignment^27^, which allows the user to align query sequences with the PlantPepDB database. This option assists the user to identify and characterize their sequence of interest. Here, we have incorporated ‘WATER’ utility of EMBOSS-6.6.0 package, following the Smith–Waterman Algorithm. ‘Mapping’ option helps the user to map all the peptide sequences of the PlantPepDB database on to the user-provided peptide sequences. Sequences having >99% similarity with user-provided sequences are displayed as the result of this alignment module. Here, we have incorporated BLASTp option of the BLAST software package. ‘BLAST’^28^ module is effective to find the regions of similarity between the FASTA sequences provided by the user and PlantPepDB database sequences using BLASTp with the option to change Expect value (E value). The respective ID(s) of PlantPepDB sequences producing significant alignments with the query sequences are further hyperlinked to display the detailed information of each peptide entry.

## Future Development

In the current release of PlantPepDB, there are 3848 peptide entries, a future release of this database will contain updated datasets with the incorporation of new tools and features. We are also planning to incorporate a tool that can predict the user’s query sequence properties by using the existing dataset of PlantPepDB. For this, we will use machine learning approaches to train the available dataset and select key features that can be used as descriptors of a plant peptide sequence. This feature will enhance the utility of PlantPepDB so that the user can also characterize an unknown peptide sequences. As the dataset size will increase we will also integrate API access for the users so that data extraction from the database in large amounts will be more straightforward and simpler.

## Conclusion

PlantPepDB is a web-based repository dedicated to plant peptides. It consists of 3848 manually curated plant peptide entries and various search options are provided for the user to explore the database. There are three major tools incorporated in the database (i.e. SW Align, Peptide Mapping, and BLAST) which helps the user to align their query peptide sequences with PlantPepDB sequences. This database provides information on different functional categories of plant peptides, their physicochemical properties, and tertiary structure.
